# The affordability of a healthy and sustainable diet: an Australian case study

**DOI:** 10.1186/s12937-020-00606-z

**Published:** 2020-09-30

**Authors:** Tara Goulding, Rebecca Lindberg, Catherine Georgina Russell

**Affiliations:** Faculty of Health, School of Exercise and Nutrition Sciences, Deakin University, Burwood, Australia

## Abstract

**Background/Aims:**

EAT–*Lancet* Commission’s Planetary Health Diet proposed a diet that integrates nutrition and sustainability considerations, however its affordability is unknown in many country-specific contexts, including Australia. The aim of this study is to develop a healthy and sustainable food basket modelled on the Planetary Health Diet to determine the affordability of the Planetary Health Diet basket across various socio-economic groups, and compare this affordability with a food basket modelled on the typical current diet, in an Australian setting.

**Methods:**

An Australian-specific Planetary Health Diet basket was developed for a reference household (2 adults and 2 children) modelled on the Planetary Health Diet reference diet, and compared to a previously-developed Typical Australian Diet basket. The cost of each food basket was determined by online supermarket shopping surveys in low, medium and high socio-economic areas in each Australian state. Basket affordability was determined for the reference household by comparing the basket cost to disposable income in each socio-economic group in each state. Mann-Whitney U tests then determined if there were significant differences between the median costs and the median affordability of both baskets.

**Results:**

The Planetary Health Diet basket was shown to be less expensive and more affordable than the Typical Australian Diet basket nationally, in all metropolitan areas, in all socio-economic groups across Australia (median cost: Planetary Health Diet = AUD$188.21, Typical Australian Diet = AUD$224.36; median affordability: Planetary Health Diet = 13%, Typical Australian Diet = 16%; *p* = < 0.05).

**Conclusions:**

This study showed the Planetary Health Diet to be more affordable than the Typical Australian Diet for metropolitan-dwelling Australians.

**Implications:**

These results can help to inform public health and food policy aimed at achieving a healthy and sustainable future for all Australians, including reductions in overweight/obesity rates and increased food security.

## Introduction

Global diets and food systems [1], and the populations relying on them, are experiencing major challenges in terms of both health and sustainability which are predicted to worsen – models project that if global eating patterns do not change away from the current diets characterised by excess energy, processed-meat and refined sugar consumption (particularly in high-income countries) and towards dietary patterns that are more rich in plant-based foods, half of the adult population and one-third of the total population (including children) will be overweight or have obesity by 2030 [2]. Current global food systems jeopardise climatic balance and ecosystem adaptability, as well as contribute to an estimated 11 million preventable adult deaths per year [3]. In order for the projected 2050 global population of 10 billion people [2] to have sufficient food to meet nutritional needs within the limits of the planet’s resources, the ways in which food systems operate must change, including which food is consumed and by whom [2–5]. Recently released research has proposed a global diet which, if widely adopted (within the context of each country and culture), is predicted to help to alleviate these issues of malnutrition and unsustainability [3].

### The inherent link between food systems and climate change

Food insecurity [6] is being exacerbated by climate change, with temperature changes, droughts and/or floods affecting food crops and consequently food accessibility in regions worldwide, including Australia [6–10]. Increased carbon dioxide in the atmosphere also contributes to a reduction in the nutrient content of food [11–13], which could have widespread health implications for the global population, in particular those who are already struggling to consume enough quality food to meet their nutritional needs [6, 13].

The extent to which climate change will affect future food security remains uncertain [7, 8], however, what is known is that while climate change affects food systems (e.g. in regard to the food able to be produced and the nutritional quality of this food), food systems also affect climate change (e.g. meat from ruminant animals contributing methane to greenhouse gas emissions) [3, 14], due to their mutually dependent relationship [8, 14–17]. Indeed, global agriculture and food production accounts for 19–29% of greenhouse gas emissions [18], 70% of freshwater use [19], ≈40% of land use [20], 78% of eutrophication [21], and 94% of the biomass of non-human mammals is livestock [21]. Together, this makes agriculture and food production one of the largest causes of environmental damage [22] which has a great effect on human and planetary health, but is also an area that we have a degree of control over to bring about positive change [3, 23]. In the EAT–*Lancet* Commission report [3], Willett et al. describe a Great Food Transformation that is predicted to result in healthier diets from sustainable food systems, for the benefit of the entire population and the planet. The need to transition to a more healthy and sustainable diet is echoed by organisations such as the Food and Agriculture Organization of the United Nations [4, 6, 24] and the Food Climate Research Network [25, 26]. The EAT–*Lancet* Commission report provides some evidence that the most effective way to lessen the environmental impact of our food systems may be to change our diet to a more sustainable one, such as the Planetary Health Diet (PHD) discussed further below [3, 27].

### The Planetary Health Diet – both healthy and sustainable

A healthy and sustainable diet has been defined elsewhere but essentially is considered to be a diet that has low environmental impact while contributing to food security and meeting the health and nutritional needs of current and future generations [3, 4, 23, 25, 28–36]. The Australian Dietary Guidelines (ADG), which have been criticised as having a reductionist approach to diet, consider nutrients first and foremost, not sustainability [37–39]. Hence, this may not be the diet to propose as optimal, especially given the demands on the food system of the consumption of the amount of meat recommended in the ADG (ruminant meat in particular is a large contributor to greenhouse gasses due to the animals methane output) [3, 14]. Australians generally consume a diet that is neither healthy nor environmentally sustainable [40, 41], though to date few countries have adopted environmental sustainability as a focus in their dietary recommendations. In contrast, Sweden and Brazil are examples of countries who have already incorporated sustainability into their dietary guidelines by including recommendations such as a predominantly plant-based diet based on seasonal and local foods, reducing food waste, and reducing consumption of red and processed meat, ultra-processed foods, and sugar-sweetened beverages [24, 42, 43].

The EAT–*Lancet* Commission’s report [3] was the first to comprehensively integrate the nutritional needs of individuals with planetary sustainability principles into a single set of global dietary recommendations. The PHD reference diet [44] is an example of a diet that is both healthy and sustainable. This reference diet forms the framework of the PHD recommendations and can be customised to regional cultural preferences [3]. The PHD reference diet was analysed as being nutrient-sufficient, and modelling showed that the intake of most nutrients increased after adoption of this diet compared with current consumption patterns, with the exception of vitamin B12 which needs fortification or supplementation [3], consistent with the current general consensus on mostly plant-based diets [23, 28, 30, 45]. The EAT–*Lancet* Commission report stated that a global shift in dietary behaviours to align with the PHD could prevent around 19–23% of deaths per year (around 11 million deaths prevented) by way of improved human health [3], however under subsequent further analysis it appears that these prevented deaths may be purely the result of the changes in energy consumption associated with the PHD [46].

### Affordability as a factor affecting food choices

For the PHD to be widely adopted, it needs to be acceptable to consumers. While there are several factors that affect consumer food choices, such as accessibility, availability, health concerns and food preferences [2, 47], this review considered purely the role of affordability as a key factor that may influence the uptake of the PHD, while acknowledging there are many other factors that also influence food choices [48]. Cost is generally a major determinant of food choices [49–57] and, although health and sustainability are desired outcomes of consumer choices, affordability often takes priority, particularly for lower-income consumers [49, 50, 58–60]. Therefore, it is necessary to understand the cost and affordability of a healthy and sustainable diet, such as the PHD, for a range of socioeconomic groups.

### Is a healthy and sustainable diet affordable for Australians?

Presently, information about the affordability of healthy and sustainable diets is scant. Only one study appeared to exist on the affordability of a healthy *and* sustainable food basket across various socio-economic groups in an Australian context (finding an increased cost to purchase the healthy and sustainable basket) [51], but this was not undertaken nationally and the basket did not include all of the sustainability principles incorporated in the PHD such as land use, nitrogen cycling, and phosphorous cycling (the EAT–*Lancet* Commission’s report was generally more comprehensive and developed specifically to help achieve the United Nations Sustainable Development Goals and Paris Agreement) [3]. Studies also exist that have been undertaken in small regions in Australia such as specific metropolitan areas [51, 61, 62], but not nationally, meaning results cannot be applied to all areas in all states, and national comparisons between different areas in different states is not possible. To our knowledge, a healthy and sustainable food basket based on the PHD has not been created and analysed for affordability nationally across various socio-economic groups in Australia. Country-specific studies are of importance due to the different cultures, customs and food availability in individual countries, as well as differing environmental factors in each country [63, 64]. This is essential for measuring the affordability, and therefore the feasibility, of a healthy and sustainable diet for all Australians. Globally, two studies from United Kingdom have determined the cost of a healthy and sustainable diet and compared it to the typical diet consumed in that country (both finding there was no cost increase to follow a healthy and sustainable diet) [65, 66]. Since the present study was completed, other research on the affordability of the PHD throughout the world has since been published, finding that the PHD was affordable for high-income countries such as Australia, but unaffordable for low-income countries [67].

The aims of this study were to: (a) Determine the affordability of the PHD food basket for low, middle and high socio-economic groups in metropolitan Australia; (b) Determine if the PHD food basket is more or less affordable than the Typical Australian Diet (TAD) food basket for low, middle and high socio-economic groups in metropolitan Australia.

## Method

### Study design

This cross-sectional study developed food baskets for a reference family of four. Food basket surveys were then conducted at Coles supermarket [68] representing the PHD and the TAD baskets (Coles and Woolworths together account for around 80% of the total grocery spend in Australia) [69], to cost the baskets in metropolitan postcodes that vary in socio-economic status, for each Australian state/territory. Metropolitan areas were chosen due to the majority of Australians (71%) dwelling in these areas [70]. The baskets were then analysed using existing secondary data from the Australian Bureau of Statistics (ABS) [71] on area level (dis)advantage and median incomes of those areas to determine affordability.

### Data collection

The reference household represents a common Australian household structure to establish the quantity of food items needed in a food basket [72]. In this study, a family of two adults (19–60 years) and two children (boy 15 years, girl 4 years) was chosen to allow for comparison to other food basket studies using the same reference household [40, 51, 61]. Additionally, the 2016 census reported that the ‘typical Australian’ (i.e. 38 years old, born in Australia of English ancestry) lives as a married couple with two children, making this household structure a sensible and representative choice [73].

To compare across various socio-economic groups, data from the ABS Socio-Economic Indexes for Areas – Relative Socio-economic Advantage and Disadvantage (SEIFA-IRSAD) was used [74]. To cover a wide range of socio-economic groups, one survey area from SEIFA-IRSAD quintile 1 (most disadvantaged), quintile 3 (no real (dis)advantage) and quintile 5 (most advantaged) from each state/territory was selected. Within each quintile in each state/territory, survey areas were defined by postcodes. Postcodes chosen were the median-ranked postcode in each state/territory (Australian Capital Territory was included in New South Wales), and non-metropolitan postcodes were excluded.

The resulting list of survey areas was composed of one postcode in each of three SEIFA-IRSAD quintiles in each of the state/territory capital city metropolitan areas in Australia (Darwin, Sydney, Melbourne, Brisbane, Adelaide, Perth and Hobart).

An assortment of food items from each category listed in the PHD reference diet [44] were selected, informed by the options proposed by Friel, Barosh and Lawrence as being both healthy and sustainable [75]. The items selected enabled sufficient consumption for the reference household for 7 days, were commonly known brands/varieties (as decided at the discretion of lead author), widely available in Australian supermarkets, and allowed for dietary variation over 7 days (the nutritional requirement of the PHD has already been established in the EAT Lancet report) [3]. The basket contents were analysed using FoodWorks v9 [76] software to ensure that the amount and energy intake in each category matched the PHD reference diet [44] as closely as possible.

The PHD reference diet [44] recommended an intake of 1323.8 g of food per adult per day, providing energy of 10,472 kJ. As the reference household used in this study comprises two adults and two children, the basket contents were increased to reflect this. The estimated energy requirements of the 15 year old boy is 12,600 kJ and of the 4 year old girl is 6100 kJ, determined using Nutrient Reference Values [77] using a physical activity level of 1.8 (moderate). Therefore, the total estimated energy requirements of the two children is 18,700 kJ, which is 89% of the combined intake of the two adults (20,944 kJ). Hence, the PHD basket was developed using the daily per-adult quantities in the reference diet [44], then multiplying by two to arrive at the basket contents for both adults, then multiplying by 1.89 to increase the basket contents by 89% to include the children’s needs, and then multiplying by seven to arrive at the final weekly basket amount.

For comparison to the usual diet consumed by Australians, the TAD basket previously developed by Friel, Barosh and Lawrence [41] was used. This pre-established food basket was based on actual consumption habits over 7 days for a reference household of two adults (19–60 years) and two children (boy 15 years, girl 4 years) as determined by national nutrition survey data [40, 41]. The household structure used for the TAD basket was the same as for the PHD basket, allowing for clear comparison. Following construction of the two baskets, each was costed to determine affordability. Additional file 1 shows both the newly-developed PHD basket and the existing TAD basket. The PHD basket matched the PHD reference diet [44] in regards to the quantity of food and energy intake.

Costing was determined using online shopping pricing data from Coles supermarkets [68] to build a hypothetical order of the basket contents to determine the cost of the food items. As Coles Online uses the same pricing for online sales as the store from which the order will be delivered from or collected [68] (confirmed via Coles Customer Care phone call, 21 May 2019), using this online pricing gives an accurate representation of prices as if the basket was purchased in store at one of the 21 postcodes selected. The survey was conducted 14th–15th August 2019.

The cheapest item available for each food item in the food basket was selected, including generic brands and temporarily out of stock items (which were assumed to be otherwise available). The item of the same size/quantity as the food basket item was selected. If there was no item of the same size, a larger size was selected and only the cost of the food basket portion was calculated and included in the basket cost on a unit-cost basis. Only non-sale prices were used.

The collection store entered into the Coles Online website was the same postcode as each survey area to capture the prices from the Coles store that residents of that postcode would likely frequent. In the event there was no Coles store in the survey area postcode, the closest store in a nearby postcode was used. The same food basket contents were used for each survey area and only the collection store changed, to determine the price of the same food basket items in each survey area. If the same item was not available in a particular store, the closest matching item was chosen. If there was no closest matching item available, the price of the item in the nearest survey area was used.

To determine the affordability of the PHD and TAD baskets, income data was required. The median family income in the postcode survey areas was determined using ABS Census data [78]. Family income data was used, as only family members are included and this calculation does not include non-family households such as group or lone households [79]. As the Census median family income data is the total income before tax, an estimate of tax paid and therefore resulting disposable income was performed using an online calculator from the Australian Taxation Office [80].

### Affordability of Planetary Health Diet and Typical Australian Diet baskets across socio-economic groups

Affordability of both baskets was calculated and compared for each socio-economic group in the survey areas using the formula *Affordability* = *Cost*÷*Income*x100, rounded to the nearest whole percent.

### Statistical analysis

Data were analysed using SPSS v23.0 [81], checked for errors, and outliers were included as the 5% trimmed mean values were very similar to the mean values. Tests of normality showed the data was non-parametric, therefore a Mann-Whitney U test was used to determine if there was a significant difference between the median costs of both baskets, and the median affordability of both baskets, using *p* < 0.05 for statistical significance. Assumptions for the Mann-Whitney U test were met for both tests.

Further, mean cost and affordability were determined for each food basket in each SEIFA-IRSAD quintile in each metropolitan area.

## Results

### Cost and affordability of the Planetary Health Diet and Typical Australian Diet baskets for low, middle and high socio-economic groups in metropolitan Australia

Table 1 details PHD and TAD weekly basket costs and affordability. While the weekly cost of each basket was the same across all SEIFA-IRSAD quintiles for each metropolitan area (i.e. Coles did not vary their prices as postcodes were changed, within areas), the weekly family disposable income varied, meaning the proportion needed to purchase each basket was greater in the lower socio-economic areas (SEIFA-IRSAD quintile 1), and lesser in the higher socio-economic areas (SEIFA-IRSAD quintile 5). At the time of the survey, the weekly cost of the PHD basket was highest in Brisbane metropolitan area ($196.60) and lowest in Sydney metropolitan area ($182.31), with affordability highest in SEIFA-IRSAD quintile 5 of Darwin and Sydney metropolitan areas (9% of weekly disposable income) and lowest in SEIFA-IRSAD quintile 1 of Darwin metropolitan area (24% of weekly disposable income). The PHD basket required a much higher proportion of income in Darwin metropolitan area SEIFA-IRSAD quintile 1 than in other survey areas, and was a statistical outlier in the data set. A comparison of the cost and affordability of PHD and TAD baskets is discussed further below. As a national average, the lowest SEIFA-IRSAD quintile required 17% of the weekly family disposable income to purchase the PHD basket compared to the highest SEIFA-IRSAD quintile at 11%. Affordability for the PHD basket was highest in Darwin and Hobart metropolitan areas (15%), and lowest in Sydney, Melbourne, Adelaide and Perth metropolitan areas (13%).

**Table 1:** Family disposable income, basket costs, and affordability per week per postcode per metropolitan area quintile

Metropolitan area and state	SEIFA-IRSAD quintile	Post code	Weekly family disposable income	Coles store	Weekly basket cost		Affordability as % of weekly income	
					Planetary Health Diet	Typical Aust. Diet	Planetary Health Diet	Typical Aust. Diet
Darwin NT	Q1	0822	$800	Coolalinga 0839^a^	$192.58	$221.68	24%	28%
	Q3	0812	$1806	Northlakes, Marrara	$192.58	$221.68	11%	12%
	Q5	0820	$2104	Darwin CBD 0800^a^	$192.58	$221.68	9%	11%
Sydney NSW/ACT	Q1	2195	$1004	Roselands 2196^a^	$182.31	$225.89	18%	22%
	Q3	2750	$1528	Penrith	$182.31	$225.89	12%	15%
	Q5	2022	$2116	Bondi Junction	$182.31	$225.89	9%	11%
Melbourne VIC	Q1	3022	$1224	Derrimut Village 3030^a^	$185.58	$220.43	15%	18%
	Q3	3173	$1416	Keysborough	$185.58	$220.43	13%	16%
	Q5	3183	$1822	Prahran 3181^a^	$185.58	$220.43	10%	12%
Brisbane QLD	Q1	4205	$1142	Waterford 4133^a^	$196.60	$224.66	17%	20%
	Q3	4127	$1404	Springwood	$196.60	$224.66	14%	16%
	Q5	4130	$1728	Loganholme 4129^a^	$196.60	$224.66	11%	13%
Adelaide SA	Q1	5115	$1212	Munno Para	$187.19	$221.61	15%	18%
	Q3	5118	$1422	Gawler	$187.19	$221.61	13%	16%
	Q5	5157	$1616	Blackwood 5051^a^	$187.19	$221.61	12%	14%
Perth WA	Q1	6064	$1280	Alexander Heights	$191.93	$234.01	15%	18%
	Q3	6057	$1570	High Wycombe	$191.93	$234.01	12%	15%
	Q5	6152	$1794	Karawara	$191.93	$234.01	11%	13%
Hobart TAS	Q1	7030	$1138	Bridgewater	$188.21	$224.36	17%	20%
	Q3	7026	$1294	Sorell 7172^a^	$188.21	$224.36	15%	17%
	Q5	7052	$1512	Kingston 7150^a^	$188.21	$224.36	12%	15%

^a^No Coles store in the selected postcode, survey taken from nearest store

### Comparison of weekly cost and affordability of the Planetary Health Diet and Typical Australian Diet baskets

As shown in Table 1 above, the weekly cost of the TAD was highest in Perth metropolitan area ($234.01) and lowest in Melbourne metropolitan area ($220.43), with affordability highest in SEIFA-IRSAD quintile 5 of Darwin and Sydney metropolitan areas (11% of disposable income) and lowest in SEIFA-IRSAD quintile 1 of Darwin metropolitan area (28% of disposable income). The TAD basket weekly cost of $234.01 was a statistical outlier for all areas in WA. Additionally, the TAD basket required a higher proportion of income (28%) in Darwin metropolitan area SEIFA-IRSAD quintile 1 than in other areas, and was also a statistical outlier.

**Fig. 1:**
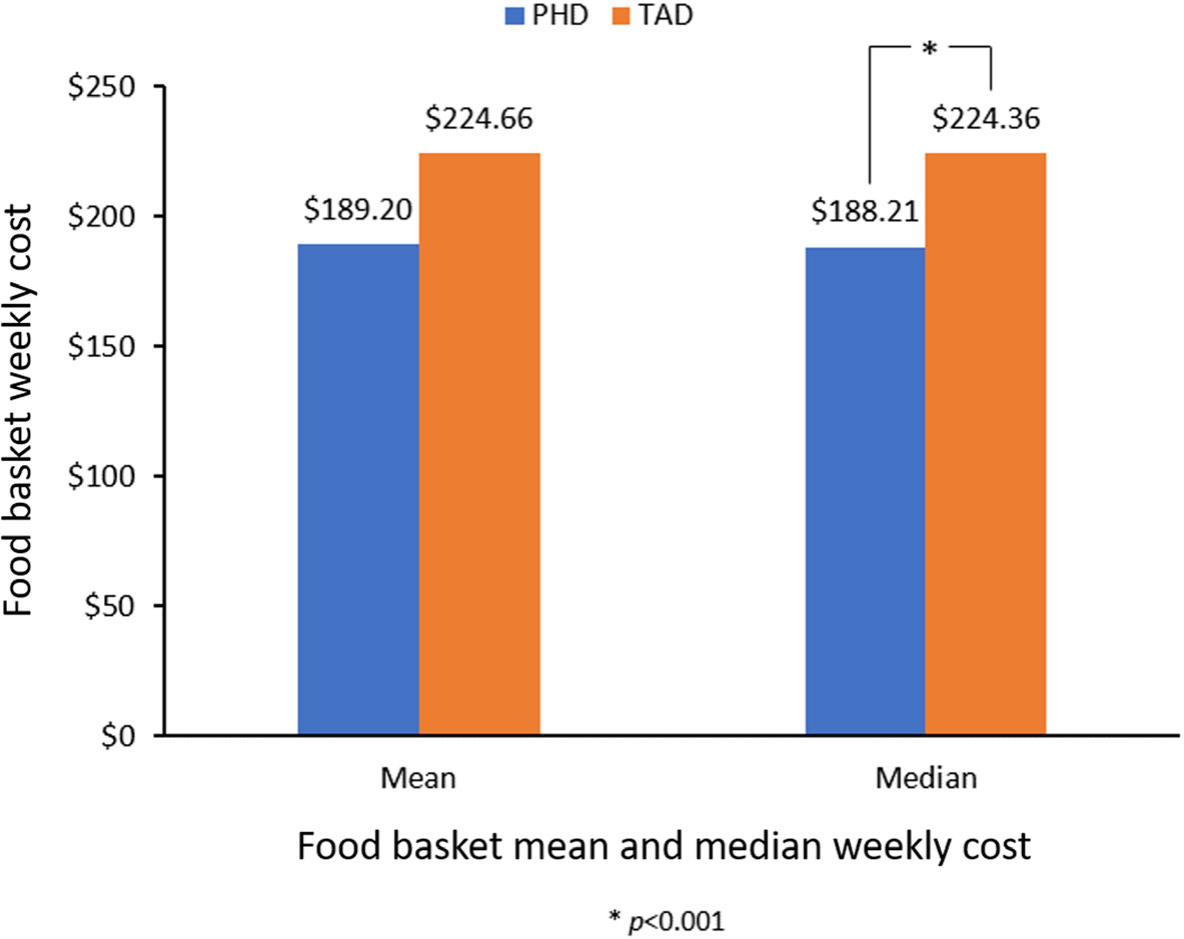
National mean and median food weekly basket cost

Figure 1 shows mean and median weekly costs of the PHD and TAD baskets nationally, demonstrating that the PHD costs less than the TAD (median is provided due to statistical test used). A Mann-Whitney U Test [81] on the median weekly costs of both baskets revealed that the PHD basket was significantly less expensive (*Md* = 188.21, *n* = 21) than the TAD basket (*Md* = 224.36, *n* = 21), *U* = 0.000, *z* = − 5.559, *p* < 0.001, *r* = − 0.86. The TAD basket also had a larger mean rank (32.00) than the PHD basket (11.00).

**Fig. 2:**
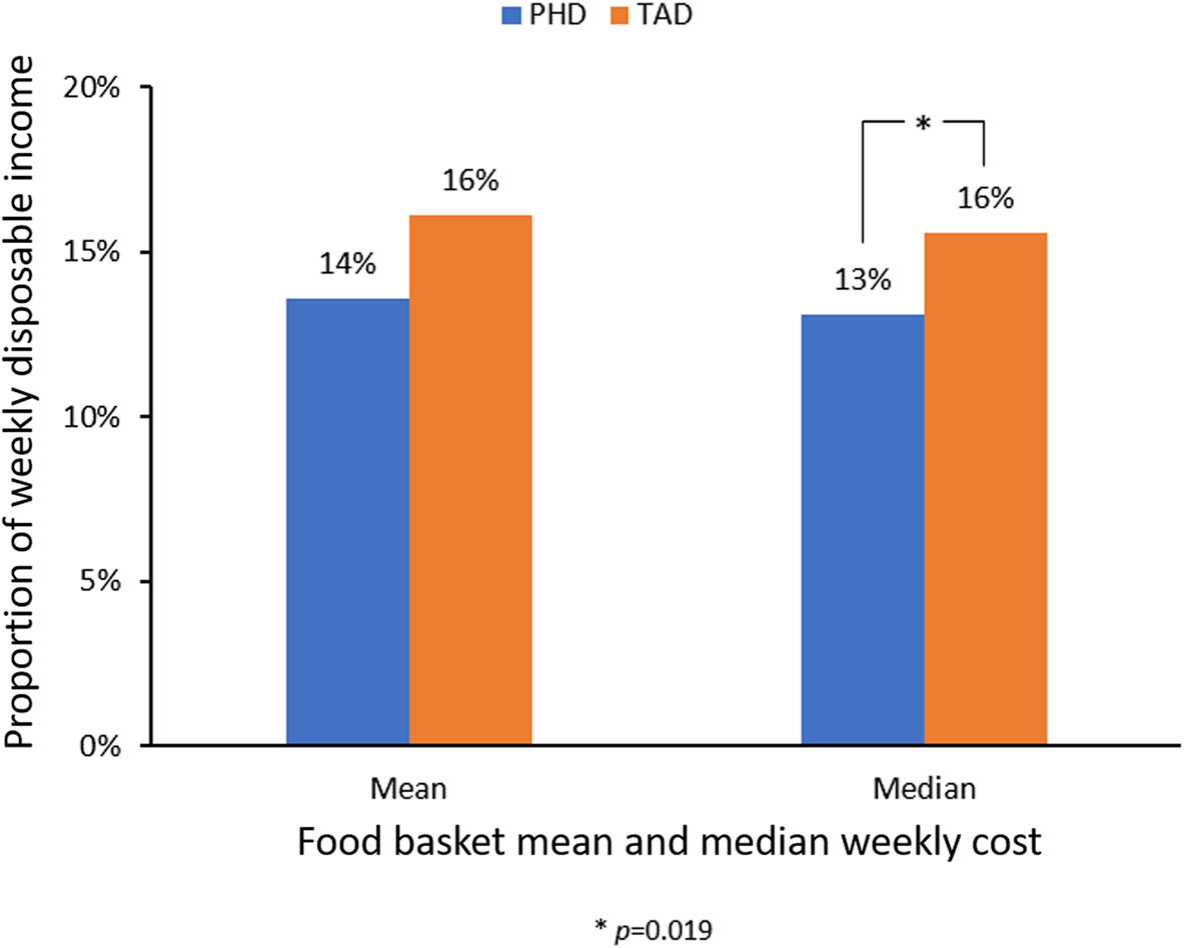
National mean food basket affordability as proportion of weekly disposable income

Figure 2 demonstrates the PHD is more affordable than the TAD (median is also provided due to statistical test used). A Mann-Whitney U Test [81] on the median affordability of both baskets revealed that the PHD basket (*Md* = 13, *n* = 21) was significantly more affordable than the TAD basket (*Md* = 16, *n* = 21), *U* = 128.00, *z* = − 2.340, *p* = 0.019, *r* = − 0.36. The TAD basket also had a larger mean rank (25.90) than the PHD basket (17.10).

**Fig. 3:**
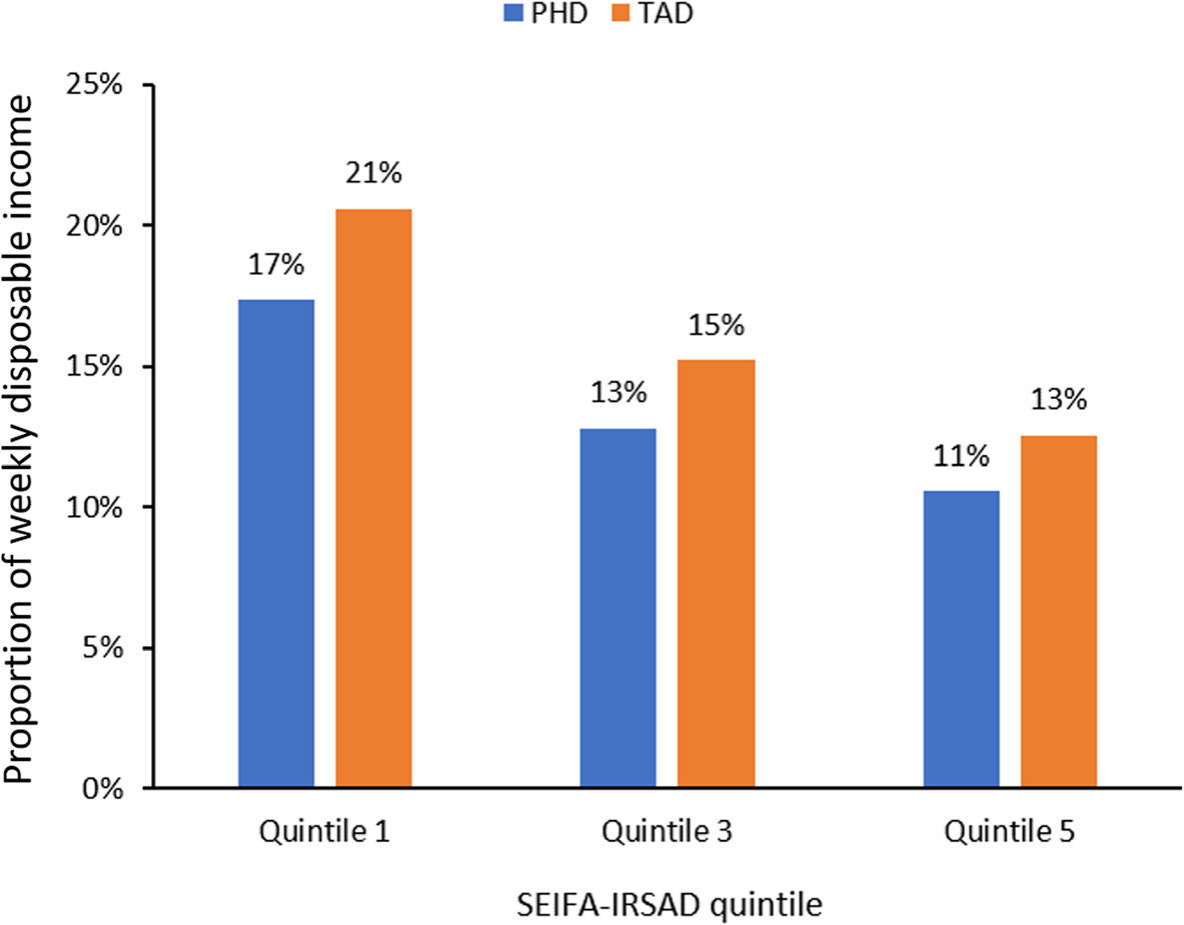
National mean food basket affordability by SEIFA-IRSAD quintile

Figure 3 shows the comparison between affordability of PHD and TAD baskets across SEIFA-IRSAD quintiles, showing that the PHD basket was more affordable (i.e. required less weekly disposable income) than the TAD basket in all quintiles nationally.

The PHD basket was less expensive than the TAD basket in all metropolitan areas across Australia, with the biggest gap in the Sydney metropolitan area supermarkets where the PHD basket was over $43 per week less expensive than the TAD basket. Consequently, the PHD basket was more affordable than the TAD basket in all state metropolitan areas.

## Discussion

The novel PHD basket for Australians was found to be both less expensive and more affordable than the TAD basket nationally, in all metropolitan areas, and across all SEIFA-IRSAD quintiles. This research suggests that healthy and sustainable diets such as the PHD are highly feasible in the Australian context, in terms of the basket contents being potentially affordable, available and purchased in large-scale retail outlets in metropolitan settings.

Results indicated that an average of AUD$189.20 (≈USD$120) per week was required to consume a diet consistent with the PHD, compared to an average of AUD$224.66 (≈USD$145) per week for the TAD. If adopted over a 1 year period, the PHD would result in savings of AUD$1843.92 (≈USD$1200) per year to the household food budget for a family of two adults and two children. This study also found SEIFA-IRSAD quintile 1 households are required to dedicate an average of 17% of their weekly income to a healthy and sustainable diet, compared to 21% of their weekly income required for a typical diet, indicating that the PHD would be more affordable for metropolitan-dwelling Australians than what was typically consumed currently regardless of socio-economic (dis)advantage.

The national mean food basket affordability results from this study were consistent with ABS data showing Australians spend approximately 17% of their disposable income on food and non-alcoholic beverages [82, 83] – in other words, the results of this study’s surveys fell into the expected range. All SEIFA-IRSAD quintiles in all metropolitan areas were within the acceptable range of food affordability (not more than 30% of disposable income) [72, 84–87], however the Darwin SEIFA-IRSAD quintile 1 survey area was nearing the domain of potential food stress for both the PHD and TAD baskets [51, 72, 85], with the baskets costing 24% and 28% of disposable income respectively.

Results of the present study are in contrast to a similar Australian study by Barosh et al. which found that a healthy and sustainable diet is more expensive than the TAD [51]. However, the disparity in results could be explained by differing methods – the surveys for the present study were conducted only in major supermarkets (Coles and Woolworths together account for around 80% of the total grocery spend in Australia) [69], whereas Barosh et al. surveyed food price data from a variety of retail outlets including small corner stores, which the authors indicated are more expensive than supermarkets [51]. In addition, the Barosh et al. healthy and sustainable basket was composed of different items to the PHD basket, and included more meat which would inevitably increase the cost of the basket, especially from smaller retail outlets [51]. Further, the Barosh et al. food basket surveys were conducted in 2011, and food costs may have changed since that time. Two studies from United Kingdom also showed that a healthy and sustainable diet cost the same or less than the typical current United Kingdom diet, consistent with the present study’s findings even though different methods were used (one study collected food costs of mid-range items from supermarkets [66], and one study collected food costs from all retail sources from consumer’s actual purchases) [65]. Additionally, Hirvonen et al. also concluded that the PHD was affordable in high-income countries such as Australia, also consistent with the present study’s findings [67].

A 2013 global review on food prices and affordability showed that for some consumers price is more of a purchasing determinant than taste, and that consumers generally purchase more food when prices fall and less food when prices rise [54]. Although the affordability of the PHD for metropolitan-dwelling Australians shopping at major supermarkets has now been demonstrated, it remains unclear if, and to what extent, the cost of fluctuating food prices could affect consumer adoption of this diet, particularly lower-income consumers who are more price sensitive than higher-income consumers [56]. This could become more of an issue in the future as it is predicted that food prices will rise due to issues associated with climate change, which will affect affordability and therefore accessibility of food for many people [2, 9].

The results of the present study suggest that a diet modelled on the PHD reference diet is feasible in regards to the weekly cost, amounts and availability of food for a range of Australians. This PHD basket was designed to accommodate Australian food preferences, and considered item availability in Australia [75] (the only item alteration was coconut oil replacing palm oil, as palm oil is neither readily accessible nor popular in Australian cuisine). Kangaroo meat was considered for inclusion due to its relatively low environmental footprint [9, 75, 88], however as this meat is not farmed but hunted on a quota system which varies between states and is dependent on the wild kangaroo population size, the supply of kangaroo meat may not be large enough or consistent enough to meet demand if the PHD is widely adopted [89]. Compared to the TAD, the total amount of meat was reduced, but most important was the reduction of ruminant meat – from 1168 g (TAD) down to 185 g (PHD). As ruminants are a large contributor to greenhouse gasses due to their methane output, this contributes greatly to the lower environmental footprint of the PHD [3, 14].

As the present results demonstrate, the PHD was less expensive and more affordable than the current TAD across socioeconomic groups. That does not mean that the PHD will be widely adopted, however this study was addressing affordability not consumer acceptability. As the PHD contains more fresh produce and no pre-prepared foods, it requires more preparation time and manual cooking than the TAD. This could be an issue for those who do not like or know how to cook, or those who are time-poor (e.g. full-time workers, single parents) and may make acceptance and compliance more difficult. The inclusion of more fresh produce also means that the basket as a whole is more perishable, hence households may need to shop more regularly than for the TAD – this could reduce feasibility of adopting the PHD for time-poor families, those who need to travel long distances to get to food retail outlets, and those who rely on public transport for travel.

The PHD basket could be made even cheaper by further reducing or even eliminating the meat portion, eating seasonally when fresh produce will be at its cheapest, buying dry goods in bulk, utilising supermarket special buys and price mark-downs, and replacing some items (e.g. fresh salmon could be replaced with less expensive tinned salmon) [68]. Likewise, the meat portion could be increased to the upper allowable limit and still be within the boundaries of the PHD diet, but this would increase the cost, as would purchasing smaller quantities and eating fresh produce out of season [68]. Therefore, the results of this study are susceptible to change based on consumer’s individual eating and shopping habits.

While this study has endeavoured to be as accurate as possible, it is not without limitations. The PHD basket contents do not consider medical dietary restrictions such as gluten-free diets for coeliacs, although this is also true for other existing food baskets [51, 61, 62, 65, 66, 90–94]. The TAD basket, while developed from surveys of actual consumption, may not be representative of an individual’s consumption, and was based on the 1995 National Nutrition Survey [40] – although the most current TAD basket available was used for this study due to time and resource constraints, future TAD baskets should be informed by the more recent 2011–13 National Nutrition and Physical Activity Survey. In addition, the present survey only used one supermarket chain so may not be representative of the cost of items purchased elsewhere, e.g. corner stores, farmers markets or grown at home. The survey also does not consider that consumers’ food choices may be influenced by sale prices, which could alter both the contents and the cost of the baskets [49–60]. The PHD basket cost did not consider varied household sizes, structures or incomes, or eating outside the home such as restaurant meals (which accounts for 34% of Australian total food expenditure) [95], although this was also true for the TAD basket therefore the comparison between the two baskets is still appropriate. In regards to taste preferences, the substantial reduction of meat in the PHD may be an obstacle for many consumers, even considering the potential financial savings associated with the PHD diet. Despite these limitations, this study still provides a worthwhile and novel contribution to the literature regarding healthy and sustainable food baskets, and the affordability of these food baskets in Australia.

### Strengths and contributions to literature

To our knowledge, this study is the first time that a food basket has been developed that is modelled on the PHD, contributing to current literature on both development and use of food baskets, and healthy and sustainable diets for different population groups. Additionally, to our knowledge, this study is the first time that a PHD basket has been costed for affordability in Australia, and that food baskets in general have been costed for affordability nationally across various socio-economic groups. This cost and affordability analysis of the PHD basket fills a gap left by the EAT–*Lancet* Commission, which did not address the diet’s economic viability for consumers in Australia [3] (global affordability has been analysed in other studies) [67]. Further research is needed to ascertain the financial feasibility of the PHD diet in rural and remote areas of Australia where food baskets cost more than in metropolitan areas [72, 90, 92, 96], as well as constructing and costing PHD food baskets for particular dietary habits such as gluten free and vegetarian and perhaps sub-population groups such as varying employment status, family structure and cultural ancestry. This study suggests that price may not be a major hurdle to shift towards sustainable and nutritious diets, and therefore attention on other consumer behaviours and levers for change is required. Further research is needed to ascertain consumer acceptance of the types and quantities of various foods that should be included in a healthy and sustainable diet, in particular the Planetary Health Diet or similar, and to determine the best method to facilitate this dietary change for all Australians.

### Implications

This study has shown that the PHD is a potentially affordable, and therefore financially feasible, diet for metropolitan-dwelling Australians regardless of socio-economic status or location. These results can help to inform public health and food policy aimed at achieving a healthy and sustainable future for all Australians. This can lead to a reduction in overweight/obesity rates and subsequent non-communicable diseases, and increased food security in the face of predicted population increases and environmental uncertainty due to global climate change effects. These results add to the available evidence used to promote food and nutrition literacy for Australians, and consumers may transition their dietary behaviour towards the PHD if not to be healthier and more environmentally friendly, then perhaps for financial benefits. For example, the existing Health Star Rating System [97] which currently rates the nutritional profile of food items, could potentially include a comparison of sustainability profiles to help consumers choose food items which fit into the framework of the PHD; or perhaps a new “Planetary Health Diet” or sustainability logo could be developed and used on PHD-compliant food items to encourage consumers to choose wisely. Given the likely increased time and cooking skills required by consumers to adopt the PHD, creating and supporting education campaigns based around food preparation and cooking skills would also be needed.

## Conclusion

This study showed that a diet modelled on the latest proposal for a healthy and sustainable diet, the PHD, is achievable within Australian food availability, cheaper when shopping at major retail outlets, and more affordable than the current Australian diet.

## Supplementary information

**Additional file 1.** Planetary Health Diet basket and Typical Australian Diet basket. Shows the food basket developed and modelled on the PHD and the existing TAD food basket [41]. The PHD basket matches the PHD reference diet [44] in regards to the quantity of food and energy intake.

## Data Availability

To be confirmed.
