# Supplementary material for: The affordability of a healthy and sustainable diet: an Australian case study
Source: Nutr J. 2020 Sep 30;19:109. doi: 10.1186/s12937-020-00606-z (PMC7528590; doi:10.1186/s12937-020-00606-z)
Supplement: Supplementary file 1 — Additional file 1. Planetary Health Diet basket and Typical Australian Diet basket. Shows the food basket developed and modelled on the PHD and the existing TAD food basket [41]. The PHD basket matches the PHD reference diet [44] in regards to the quantity of food and energy intake. [file 12937_2020_606_MOESM1_ESM.docx]

## Planetary Health Diet basket and Typical Australian Diet basket^41^

Based on the average weekly intake of a household of four: adult male (aged 19-60 years), adult female (aged 19-60 years), boy aged 15 years and girl aged 4 years.

| **Planetary Health Diet basket** | | | **Typical Australian Diet basket^41^** | | |
| --- | --- | --- | --- | --- | --- |
| **Basket item** | **Amount** | | **Basket item** | **Amount** | |
| **Whole grains** |  |  | **Vegetables** |  |  |
| Rice, brown, uncooked | 1323 | g | Carrots (pre-packed) | 225 | g |
| Bread, mixed grain & seeds, wholemeal, extra grainy, other seeds, fresh | 185 | g | Cauliflower (pre-packed) | 487.5 | g |
|  |  |  | Potatoes (pre-packed) | 352 | g |
| Rolled oats, uncooked, plain, unfortified | 1191 | g | Tomatoes (pre-packed) | 720 | g |
| Pasta, wholemeal, dry | 1191 | g | Lettuce (pre-packed) | 1800 | g |
| Barley, uncooked | 1058 | g | Mushrooms (pre-packed) | 296 | g |
| Quinoa, uncooked | 1191 | g | Onion (pre-packed) | 315 | g |
| **Tubers or starchy vegetables** |  |  | Frozen mixed veg | 1160 | g |
| Potato, plain, other, other, unpeeled, raw | 1323 | g | Pumpkin | 70 | g |
| **Vegetables - dark green** |  |  | Sweet potatoes (pre-packed) | 560 | g |
| Broccoli, fresh, raw | 556 | g | Tinned green beans | 1980 | g |
| Kale, raw | 476 | g | Zucchini (pre-packed) | 1179 | g |
| Baby spinach, raw | 318 | g | **Fruit** |  |  |
| Cabbage, savoy, raw | 397 | g | Green apples (pre-packed) | 624 | g |
| Lettuce, cos | 212 | g | Peaches (pre-packed) | 1240 | g |
| Capsicum, green, fresh, raw | 212 | g | Red apples (pre-packed) | 1480 | g |
| Zucchini, green, fresh, unpeeled, raw | 265 | g | Tinned fruit salad | 1020 | g |
| Cucumber, common, unpeeled | 212 | g | Dried sultanas | 340 | g |
| **Vegetables - red and orange** |  |  | **Grain (cereal) foods** |  |  |
| Capsicum, red, fresh, raw | 265 | g | Cornflakes | 594 | g |
| Carrot, regular, fresh, unpeeled, raw | 582 | g | Muesli | 2880 | g |
| Pumpkin, butternut, fresh, raw | 529 | g | White bread (pre-packed) | 6080 | g |
| Sweet potato, orange, plain, unpeeled, fresh, raw | 132 | g | Turkish bread (pre-packed) | 340 | g |
|  |  |  | White rice | 8200 | g |
| Tomato, common, raw | 1138 | g | White flour | 33.6 | g |
| **Vegetables - other** |  |  | White pasta | 520 | g |
| Mushroom, fresh, common, fresh | 1879 | g | **Meats and poultry, fish, eggs, tofu, nuts and seeds, and legumes/beans** |  |  |
| Onion, mature, brown, raw | 714 | g |  |  |  |
| Garlic, fresh, raw | 53 | g | Chicken breast | 680 | g |
| **Fruits** |  |  | Beef steak | 400 | g |
| Banana, fresh, cavendish | 1323 | g | Lamb chops | 368 | g |
| Apple, fresh, pink lady, unpeeled | 1323 | g | Minced beef | 400 | g |
| Kiwifruit, green (hayward), unpeeled | 794 | g | Sliced ham | 210 | g |
| Mandarin, fresh | 1191 | g | Eggs | 472 | g |
| Strawberries, fresh | 529 | g | **Milk, yoghurt, cheese and/or their alternatives** |  |  |
| Avocado, raw | 132 | g |  |  |  |
| **Dairy foods** |  |  | Cheddar cheese | 630 | g |
| Milk, cow, ready to drink, regular fat, regular | 6422 | mL | Milk, whole | 8250 | mL |
| **Protein sources - animal** |  |  | Yoghurt | 3200 | g |
| Beef, diced, untrimmed, raw | 185 | g | **Allowance for unsaturated spreads and oils** |  |  |
| Pork, diced, raw | 185 | g | Margarine | 144 | g |
| Eggs, chicken, whole, raw, regular | 344 | g | **Discretionary food choices** |  |  |
| Fish, salmon, raw, atlantic | 741 | g | Butter | 320 | g |
| Chicken, breast, with skin, raw | 767 | g | Chicken stock | 15 | g |
| **Protein sources - plant** |  |  | Coca Cola | 2400 | mL |
| Lentils, red, dried | 926 | g | Frozen fish sticks | 320 | g |
| Beans, red kidney, dried, uncooked | 132 | g | Frozen meat pie | 1520 | g |
| Beans, cannellini, dried | 132 | g | Frozen pizza | 1040 | g |
| Peas, split, uncooked | 265 | g | Ice cream | 899 | mL |
| Tofu, firm | 132 | g | Lamington biscuit | 300 | g |
| Nuts, peanut, raw, unsalted | 1058 | g | Mayonnaise | 240 | g |
| **Tree nuts** |  |  | Orange juice drink | 3626 | mL |
| Nuts, almonds, raw, with skin | 53 | g | Potato chips | 88 | g |
| Nuts, cashews, raw | 265 | g | Sugar, white | 28 | g |
| Seeds, sunflower | 344 | g | Tinned spaghetti | 1590 | g |
| **Added fats** |  |  | Tomato soup | 880 | g |
| Oil, coconut | 196 | mL | Jam | 104 | g |
| Oil, olive, extra virgin | 1151 | mL | Popcorn | 135 | g |
| Lard | 132 | g | Worcester sauce | 20 | g |
| **Added sweeteners** |  |  |  |  |  |
| Sugar, raw, regular | 820 | g |  |  |  |
